# Low insulin-like growth factor 1 is associated with low high-density lipoprotein cholesterol and metabolic syndrome in Chinese nondiabetic obese children and adolescents: a cross-sectional study

**DOI:** 10.1186/s12944-016-0275-7

**Published:** 2016-06-24

**Authors:** Shuang Liang, Yanyan Hu, Caihong Liu, Jianhong Qi, Guimei Li

**Affiliations:** Department of Pediatrics, Shandong Provincial Hospital Affiliated to Shandong University, 9677 Jingshi Road, Jinan, 250021 China

**Keywords:** Insulin like growth factor 1, High-density lipoprotein cholesterol, Metabolic syndrome, Obesity, Children

## Abstract

**Background:**

Low serum high-density lipoprotein cholesterol (HDL-C) is an independent risk factor for developing cardiovascular disease. Insulin-like growth factor 1(IGF-1) levels have been proven to be positively associated with HDL-C, but few studies were based on the dataset of children or adolescents. The aim of this study is to investigate the relationship among IGF-1, HDL-C and the metabolic syndrome in Chinese nondiabetic obese children and adolescents.

**Methods:**

As a cross-sectional study, this study includes 120 obese Chinese children and adolescents and **120** healthy ones. The obese subjects were divided into two groups based on using 1.03 mmol/L as a threshold value for HDL-C. Clinical examination and laboratory examinations were assessed for all participants.

**Results:**

Obese subjects had significantly lower IGF-1SDS **and higher Height SDS** than those in the control group. Among 120 obese children and adolescents, 22 (18.3 %) subjects had an HDL-C level <1.03 mmol/L. IGF-1SDS was significantly lower (*P* = 0.001) in obese subjects with low HDL-C. According to the results of multivariate logistic regression analysis, IGF-1 SDS is significantly associated with low HDL-C(OR 0.518, 95 % CI 0.292–0.916; *P* = 0.024), after being adjusted for age, gender, pubertal status, BMI SDS, SBP, DBP, HOMR-IR, total cholesterol, low density lipoprotein-cholesterol, triglycerides, ALT and uric acid. In addition, IGF-1 SDS is significantly correlated with the level of serum HDL-C in study population (*r* = 0.19, *P* = 0.003). Based on logistic regression analysis with adjustment for age, gender and pubertal status, the increased IGF-1 SDS was associated with a decreased probability of metabolic syndrome (OR 0.555, 95 % CI 0.385–0.801; *P* = 0.002) and hypertriglyceridemia (OR 0.582, 95 % CI 0.395–0.856; *P* = 0.006), but no significant correlation with hypertension.

**Conclusion:**

**Obese children had lower IGF-1SDS and taller stature compared with the control group.** Low levels of IGF-1 SDS were associated with low levels of HDL-C in chinese nondiabetic obese children and adolescents, independent of insulin resistance, as well as other traditional cardiovascular disease risk markers.

## Background

The prevalence and severity of childhood obesity are increasing rapidly, and they have reached epidemic levels globally. In developed countries, the prevalence of overweight and obesity has been reported to be 23.8 % of boys and 22.6 % of girls in 2013 [1]. A previous survey conducted by Chinese scholars showed that the prevalence of obesity was 49.1 % for boys and 30.8 % for girls in the center of Shanghai city [2]. The ever-growing number of obesity children will cause lots of serious issues since obesity is recognized as a central feature of the metabolic syndrome, and it is always associated with insulin resistance, hypertension, dyslipidaemia, hyperglycemia and cardiovascular disease [3]. Low serum high-density lipoprotein cholesterol (HDL-C), which is identified as an independent risk factor for developing cardiovascular disease is also linked with obesity.

As an important mediator of growth hormone (GH) secretion, Insulin-like growth factor 1 (IGF-1) is primarily synthesized from the liver, which plays a key role in normal growth and development. Nowadays, increasing evidences suggest that a lower IGF-1 level is associated with obesity [4], insulin resistance [5], metabolic syndrome [6, 7], impaired glucose tolerance [8], nonalcoholic fatty liver disease (NAFLD) [9] and cardiovascular disease [10]. Similarly, HDL-C and IGF-1 share many common features. Both of them are secreted partially from the liver, and HDL-C is also linked to the same cluster of cardio-metabolic disorders. Moreover, the positive correlation between IGF-1 and HDL-C had been proposed in some studies [11–14]. However, previous studies relating to the relationship between HDL-C and IGF-1 are still some topical and controversial. Furthermore, this relationship didn’t focus on the samples of children and adolescents, especially those of obese group. Therefore, in this present study, we aimed to investigate the association between IGF-1 and HDL-C, metabolic syndrome in nondiabetic obese children and adolescents.

## Methods

### Subjects

This retrospective cross-sectional study was carried out at the Department of Pediatrics of Shandong Provincial Hospital affiliated to Shandong University. We investigated a group of obese subjects and a group of healthy controls respectively. A total of 120 obese children and adolescents (age ranging from 10 to 16) was conducted from July of 2011 to November of 2015. The diagnosis of obesity is those children whose body mass index (BMI) are above the 95th percentile with age and sex adjustment, based on the national reference data [15]. The exclusion criteria for our study were: 1) children who had ever had renal disease, liver failure, cancer, and other systemic severe diseases; 2) children with hypothalamus diseases, pituitary diseases, thyroid dysfunction, diabetes mellitus, chromosome abnormalities or all sorts of syndromes; 3) the obese children who had been smokers, and those who ever had chronic alcohol intake, and used drugs which may influence lipid metabolism, blood pressure, liver function, insulin action, glucose and weight. Control subjects which **include 120 children and adolescents,** were recruited from a population of non-obese healthy children and adolescents. The obesity group and control group were matched on age, pubertal status and gender. The study was approved by the Ethics Committee of the Shandong Provincial Hospital Affiliated to Shandong University. Written informed consents had been signed by all participants’ parents.

### Anthropomorphic measurements

Anthropometric measurements, including measurements of weight, **height,** systolic blood pressure (SBP), diastolic blood pressure (DBP), and pubertal staging were assessed for each participant. Body weight was measured to the nearest 0.1 kg for all subjects wearing light clothing and no shoes. The degree of obesity was calculated as body mass index (BMI) (kg/m2). To minimize the confounding effects of age and sex, we transformed BMI into BMI standard deviation scores (BMI SDS) based on normative values for Chinese children [15]. **Height was measured in the morning, and there was also nearest 0.1 cm errors. Height was expressed as height standard deviation scores (Height SDS) based on normative values for Chinese children** [16]**.** An average of three blood pressure measurements was taken while the subjects were seated after 5 min’ rest. The stage of puberty was assessed according to Tanner criteria [17]. Boys with pubic hair and gonadal stage 1, and girls with pubic hair and breast stage 1 were considered as prepuberty [18, 19].

### Laboratory measurements

Fasting blood samples were obtained for all participants after an overnight fast to measure serum IGF-1 level and other metabolic parameters.

Serum concentration of IGF-1 was estimated based on chemiluminescence assay (IMMULITE 2000, Siemens Health care Diagnostics, USA). Intra-assay and inter-assay coefficient of variation declared by the manufacturer was 2.5–7.6 % for IGF-1 measurement. IGF-1 was represented as IGF-1 standard deviation scores (IGF-1 SDS), based on age and sex normal range of the population by past researches [20]. Total cholesterol (TC), HDL-C, low density lipoprotein-cholesterol(LDL-C), triglycerides(TG), fasting plasma glucose (FPG), alanine aminotransferase (ALT), and uric acid (UA) were determined using an Auto Biochemical Analyzer (AU5400, Beckman Coulter, Tokyo, Japan). Fasting insulin were assayed using a chemiluminescent immunometric assays (CobasE170, Roche Diagnostics, Mannheim, Germany). The intra-assay and inter-assay coefficients of variation were <7.0 % in these assays. Estimates of insulin resistance were calculated using the following formula of homeostasis model assessment-insulin resistance (HOMA-IR) = insulin (uIU/ml) × glucose (mmol/l))/22.5 [21]. An oral glucose tolerance test (OGTT) was performed with 1.75 g of glucose kg of weight (to a maximum of 75 g) in fasting glucose ≥ 5.6 mmol/L subjects in order to rule out diabetes mellitus.

### Definition

The metabolic syndrome was defined as the presence of any three or more of the following five constituent risks according to the criteria of International Diabetes Federation [22]: 1) abdominal obesity. 2) low HDL-C. 3) hypertriglyceridemia. 4) hypertension. 5) impaired fasting glucose. Low HDL-C was defined as HDL-C levels <1.03 mmol/L. Hypertriglyceridemia was defined as triglyceride ≥ 1.7 mmol/L. Hypertension was defined as SBP ≥ 130 mmHg or DBP ≥ 85 mmHg. Impaired fasting glucose was defined as fasting glucose ≥ 5.6 mmol/L.

### Statistical analysis

Normally distributed variables were expressed as means ± standard deviation (SD). Skewed distributed variables were natural log transformed, and were expressed as mean ± SD. Skewed distributed variables were expressed as median (interquartile range) since they can not be transformed to normal distribution. Groups were compared using the Student’s t tests or Mann–Whitney *U* test. Categorical variables were compared by chi square test. The relationships among serum IGF-1 SDS, clinical and metabolic variables were evaluated with Pearson’s correlation analysis or Spearman correlation analysis. A multivariable logistic regression analysis was used to determine the association between IGF-1 SDS and the low HDL-C(<1.03 mmol/L) after adjustment for anthropometric, metabolic variables. In multiple logistic models, only HOMA-IR representing fasting glucose and insulin respectively, were used to avoid collinearity. Logistic regression analysis was also applied to demonstrate the association among the metabolic syndrome, hypertriglyceridemia, hypertension and IGF-1SDS. The presence of the metabolic syndrome, hypertriglyceridemia and hypertension was used as a dependent variable and IGF-1SDS as an independent variable after adjustment for age, gender and pubertal status. A *P* value <0.05 was considered statistically significant. Statistical analyses were all carried out using SPSS version 17.0 (SPSS Inc. Chicago, USA).

## Results

The characteristics of the study population are shown in Table 1. Gender, age, pubertal status and fasting glucose were similar in obesity group and control group. In the obese group, significant percentages of clinical and metabolic alterations were observed. Notably, The obese group had significantly lower IGF-1 SDS (*P* < 0.001), HDL-C (*P* < 0.001) than the control group. In contrast, the obese group showed significantly higher levels of **Height SDS**, SBP, DBP, BMI SDS, HOMA-IR than the control group, The levels of total cholesterol, low density lipoprotein-cholesterol, triglycerides, uric acid, ALT also were significantly higher in the obese group than those in the control group.

**Table 1:** Clinical and laboratory characteristics of study participants

Characteristics	Obese group (*n* = 120)	Control group (*n* = 120)	*P* value
Age (yr)	12.09(1.35)	11.63(2.27)	0.054
Male/Female, (n)	101/19	88/32	0.058^a^
Prepuberty/Puberty,(n)	81/39	76/44	0.587^a^
IGF-1SDS	−0.33(1.35)	0.66(1.23)	<0.001*
SBP (mmHg)	121.08(12.84)	106.69(9.7)	<0.001*
DBP (mmHg)	78.79(10.41)	70.69(7.61)	<0.001*
BMISDS	2.71(0.78)	0.04(0.77)	<0.001*
Height SDS	1.03(0.46–1.93)	−0.26(−0.71–0.41)	<0.001*^a^
HOMA-IR	6.35(3.8)	2.67(1.53)	<0.001*
FPG (mmol/L)	5.08(0.44)	5.07(0.42)	0.894
TC (mmol/L)	4.24(3.78–4.84)	4.01(3.64–4.37)	<0.001*^a^
TG (mmol/L)	1.4(0.87)	0.79(0.32)	<0.001*
HDL-C (mmol/L)	1.23(0.29)	1.53(0.33)	<0.001*
LDL-C (mmol/L)	2.53(0.61)	2.11(0.52)	<0.001*
UA(μmol/L)	386.68(96.18)	298.29(69.42)	<0.001*
ALT((U/L))	25.00(18.00–40.00)	13.50(11.00–17.00)	<0.001*^a^

SBP, HOMA-IR, triglycerides, uric acid were log transformed for statistical analysis, but values in the table represent a back transformation to the original

* *P* < 0.05, ^a^Mann-Whitney *U* test or the chi-square test

A total of 120 obese participants with a mean age of 12.09 ± 1.35 years old were included in the study. Among the 120 obese participants, 101 (84.2 %) children were male. The majority of children were prepubertal (81, 67.5 %). Low levels of HDL-C were observed in 22 of 120 obese participants (18.3 %). 26 subjects had a triglyceride ≥ 1.7 mmol/L(21.7 %, 26/120), while Hypertension was observed in 39.2 % (47/120) of assessed individuals and metabolic syndrome in 28.3 % (34/120) of them. In addition, we observed 18 of 120 obese participants (15 %), which has impaired fasting glucose after excluding type 1 and 2 diabetes mellitus.

Obese children were further divided into two groups according to HDL-C level. The characteristics of the two groups are shown in Table 2. IGF-1 SDS were significantly lower (*P* = 0.001) in obese subjects with low HDL-C group than in subjects with HDL-C ≥1.03 mmol/L, whereas triglycerides were higher in obese children with HDL-C < 1.03 mmol/L than obese children with HDL-C ≥1.03 mmol/L. No difference existed in the age, gender, pubertal status, **Height SDS**, SBP, DBP, BMI SDS, HOMA-IR, fasting glucose, total cholesterol, low density lipoprotein-cholesterol, uric acid and ALT between the two groups.

**Table 2:** Clinical and laboratory characteristics in the two groups of subjects using1.03 mmol/L as a threshold value for HDL-C

Characteristics	HDL-C <1.03 mmol/L (*n* = 22)	HDL-C ≥ 1.03 mmol/L (*n* = 98)	*P* value
Age (yr)	12.1(1.13)	12.09(1.4)	0.992
Male/Female, (n)	16/6	85/13	0.115^a^
Prepuberty/Puberty,(n)	14/8	67/31	0.802^a^
IGF-1SDS	−1.19(1.4)	−0.14(1.27)	0.001*
SBP (mmHg)	121.14(12.43)	121.07(13)	0.963
DBP (mmHg)	80.05(9.37)	78.51(10.65)	0.534
Height SDS	1.18(0.67–1.83)	1.02(0.3–1.97)	0.722^a^
BMISDS	2.85(0.82)	2.68(0.77)	0.34
HOMA-IR	6.37(4.05)	6.34(3.77)	0.955
FPG (mmol/L)	5.08(0.47)	5.08(0.43)	0.989
TC (mmol/L)	4.12(0.76)	4.46(0.93)	0.105
TG (mmol/L)	2.22(1.47)	1.22(0.53)	<0.001*
LDL-C (mmol/L)	2.43(0.47)	2.55(0.63)	0.393
UA(μmol/L)	399.32(95.85)	383.85(96.52)	0.491
ALT((U/L))	23.5(19.75–37.25)	26.00(17.75–40.00)	0.807^a^

SBP, HOMA-IR, total cholesterol, triglycerides, uric acid were log transformed for statistical analysis, but values in the table represent a back transformation to the original

* *P* < 0.05, ^a^Mann-Whitney *U* test or the chi-square test

Through multiple logistic regression analysis, we observed that in the final model, after adjusting for age, gender, pubertal status, BMI SDS, SBP, DBP, HOMR-IR, total cholesterol, low density lipoprotein-cholesterol, triglycerides, ALT, uric acid. Notably, the increased IGF-1SDS was associated with a decreased probability of low HDL-C (OR 0.518, 95 % CI 0.292–0.916; *P* = 0.024).

We performed a bivariate correlation analysis to explicit the relationship between IGF-1SDS and cardiometabolic variables in study population (Table 3). IGF-1 SDS was significantly correlated with HDL-C(*r* = 0.19, *P* = 0.003), BMI SDS(*r* = −0.25, *P* < 0.001), SBP (*r* = −0.134, *P* = 0.038), DBP (*r* = −0.197, *P* = 0.002), total cholesterol (*r* = −0.157, *P* = 0.015), low density lipoprotein-cholesterol (*r* = −0.182, *P* = 0.005), triglycerides (*r* = −0.179, *P* = 0.006) and ALT (*r* = −0.266, *P* < 0.001). But no correlation was observed with age, HOMA-IR, fasting glucose and uric acid. Notably, correlation between IGF-1 SDS and HDL-C is also significant.

**Table 3:** Bivariate correlation analysis between IGF-1 SDS and cardiometabolic variables in study population

Variable	r	*p*
Age (yr)	0.112	0.084
SBP (mmHg)	−0.134	0.038*
DBP (mmHg)	−0.197	0.002*
BMI SDS	−0.25	<0.001*
HOMA-IR	−0.07	0.279
TC (mmol/L)	−0.157	0.015*
HDL-C (mmol/L)	0.19	0.003*
LDL-C (mmol/L)	−0.182	0.005*
TG (mmol/L)	−0.179	0.006*
UA(μmol/L)	0	0.999
FPG (mmol/L)	0.107	0.099
ALT((U/L))	−0.266	<0.001*

* *P* < 0.05

Logistic regression analysis was also applied to study the association among IGF-1SDS and the metabolic syndrome, hypertriglyceridemia and hypertension, adjusted for gender and pubertal status. The increased IGF-1 SDS was associated with a decreased probability of metabolic syndrome (OR 0.555, 95 % CI 0.385–0.801; *P* = 0.002) and hypertriglyceridemia (OR 0.582, 95 % CI 0.395–0.856; *P* = 0.006), but with no significant correlation with hypertension (OR 0.765, 95 % CI 0.567–1.031; *P* = 0.079).

## Discussion

In this study, we found that low IGF-1 SDS is positively correlated with low HDL-C levels in nondiabetic obese children and adolescents. More importantly, the positive relationship remains significant after controlling several confounders.

Obesity represents a major worldwide health problem, and it is always associated with the incidence of multiple comorbidities. In present study, in accordance with previous reports [3], we found that obese children had significantly lower HDL-C, higher levels of SBP, DBP, BMI SDS, HOMA-IR, uric acid, ALT and an adverse lipid profile compared with the control group.

Apart from obesity-related co-morbidities, obesity is also associated with endocrine perturbations. The influences of obesity on the GH-IGF-1 axis and growth have been recognized. There are lots of evidences to suggest that obesity is associated with the reduced stimulated GH release [23, 24] as well as the endogenous GH secretion [25]. This GH deficiency associated with obesity is relative and with weight loss GH secretion is fully reversed [24]. But unlike the unequivocal effects of significantly lower GH observed in patients with obesity, there are some controversies about the relationship between obesity and IGF-1. Reduced IGF-1 [26, 27], normal [28] or high [29] IGF-1 in obese subjects have been reported previously. Accordingly, we futher assessed the relationship between IGF-1SDS and obesity, and our study showed that the obese subjects had lower IGF-1 SDS compaed with control group. The result is consistent with those of a previously epidemiological study based on 3328 subjects (aged 19–72 years) which reported a similar negative correlation between IGF-1 and obesity-related anthropometric markers in both men and women [26]. Another study based on a small sample dataset also showed that obese adolescent had lower total IGF-1 values comparing with lean adolescent subjects [27]. The plausible reasons for these differences of previous studies are that: (1) we used the IGF-1SDS as a proxy of IGF-1; (2) the different samples may creates different results due to heterogeneity.

Interestingly, despite low GH secretion and the abnormalities in the peripheral GH-IGF system in obese children, a normal or tall stature is usually observed [30]. Several studies have shown that obese children have higher height velocity and taller stature than non-obese children during the prepubertal years, while similar final heights were usually observed that between obese and nonobese children [31, 32]. Our study is mainly based on a sample of prepuberty population and the results of this study showed that obese group had higher height SDS compared with control group which is also consistent with those of previous studies. The underlying mechanisms in this paradoxical combination of the abnormalities GH-IGF axis and tall stature remain unknown. Severalmechanisms such as increased leptin [33] and insulin [30], increased amount of free-IGF-1 [27], upregulation of GH receptors [34] have been suggested.

In this study, we provided evidences that lower IGF-1 is positively associated with low HDL-C (HDL-C < 1.03 mmol/L) in Chinese nondiabetic obese children and adolescents. First, patients with lower HDL-C had lower IGF-1 SDS. Second, IGF-1 SDS was significantly correlated with serum HDL-C level. Third, logistic regression analysis further showed that lower IGF-1SDS significantly contributed to the risk of low HDL-C. Decreased IGF-1 and HDL-C levels have been reported to be associated with insulin resistance [5, 35–37]. Our results showed that decreased levels of IGF-1SDS in obese children and adolescents were associated with low HDL-C, independent of insulin resistance, as well as other traditional cardiovascular disease risk markers. Causal relationship between HDL-C and IGF-1 levels was not established in this cross sectional series. However, we speculate that IGF-1 might exert some direct or indirect effects on HDL-C metabolism.

The exact mechanisms of the relationship between IGF-1 and HDL-C in obesity are not fully understood at present. The possible reasons might be listed as following: 1) it is easy to explain and prove that IGF-1 may influence HDL-C, at least partially by improving the insulin sensitivity [38]. IGF-1 shares structural homology and downstream pathways with insulin. Low plasma IGF-1 is associated with reduced insulin sensitivity and increased insulin resistance, which had been showed in clinical studies [35, 39]. Insulin resistance was considered to affect HDL-C metabolism and result in lower HDL-C [40, 41]. 2) Another possible explanations for the association between IGF-1 and HDL-C are driven by GH. Since it is known that IGF-1 serves as a proxy for the pulsatile GH secretion. Basal and peak stimulated GH levels were positively associated with HDL-C in several research [42–44], and GH treatment in some studies had been shown to be correlated with a higher level of HDL-C [45, 46]. It supports that IGF-1 is correlated with modulating serum HDL concentration. 3)IGF-1 inhibits expression of hepatic scavenger receptor of class BI (SRB1) on the surface of liver Hep2 B cells, leading to a reduction of HDL reverse transport and an increased circulating HDL-C concentration [47].

Our study highlights the importance of IGF-1 to regulate HDL-C. As the component of metabolic syndrome, low HDL-C may be partially accounted for the association between IGF-1 and risk of metabolic syndrome. Several studies had suggested that IGF-1 had robust negative relationships with metabolic syndrome and its components [6, 12, 48–51]. Saydah. et al.[48] and Parekh. et al. [49] noted that IGF-1 concentrations were negatively correlated with metabolic syndrome as well as its individual components among NHANES III participants. In another study among patients with impaired glucose tolerance and subjects with and without diabetes, low plasma IGF-1 concentrations were significantly associated with the presence of metabolic syndrome and were inversely associated with a number of individual metabolic syndrome components [12]. However, some other clinical reports showed some inconsistent results regarding the relationship between IGF-1 with metabolic syndrome [52] and its components [53]. A possible reason for this discrepancy might be that different studies were based on the different characteristics of study groups. In Chinese nondiabetic obese children and adolescents, we observed that the association between low IGF-1 SDS and metabolic syndrome was independent of age, gender and pubertal status. We further demonstrated low IGF-1 SDS is in negative correlation with hypertriglyceridemia independent of age, gender and pubertal status, but it was not in statistically significant association with hypertension. This is the first study in obese children concerning IGF-1 and metabolic syndrome and its components.

The positive association between low IGF-1 with a low level of HDL-C and metabolic syndrome observed in this study suggests that a low IGF-1 level contributes to the increased risk of cardiovascular disease in obese children and adolescents, implying that IGF-1 may be an important biomarker for clinicians to identify subjects at risk for early cardiovascular disease in obese children. Further research in prospective studies is needed to better understand these relationships.

The present study has several strengths and fills some research gaps. First, to the best of our knowledge, our study is one of the first to focus on the relationship between circulating IGF-1 levels and serum HDL-C based on a dataset of obese children individuals independent of several confounding factors. Second, several confounding variables had been reported to affect IGF-1 levels. In the current study, in order to limit the confounding effects of age and sex, we used the IGF-1 SDS as a proxy of IGF-1. The standardized IGF-1 levels have not been applied in previous studies regarding the relationship between IGF-1 and HDL-C. Third, our study included the relatively large sample size with detailed clinical characterization.

However, our study has some potential limitations. The first limitation is the cross sectional design of this study doesn’t not allow us to determine causality. Secondly, we did not analyze GH, IGF binding proteins (IGFBPs) and free IGF-1. Thirdly, serum HDL-C and IGF-1 were measured without replication.

## Conclusion

In conclusion, our results suggest that, **obese children and adolescents had lower IGF-1SDS and taller stature compared with the control group**. There is an independent association among lower IGF-1, low HDL-C and metabolic syndrome. Thus, the inclusion of measurement of IGF-1 and HDL-C levels might be warranted in the assessment protocols for obese or overweight children and adolescents, in order to verify possible complications of early cardiovascular alterations. Also developing a new prevention and treatment strategies for increasing serum IGF-1 levels would benefit in controling low HDL-C and metabolic syndrome.

## Abbreviations

ALT, alanine aminotransferase; BMI SDS, BMI standard deviation scores; BMI, body mass index; DBP, diastolic blood pressure; FPG, fasting plasma glucose; GH, growth hormone; HDL-C, high-density lipoprotein cholesterol; Height SDS, height standard deviation scores; HOMA-IR, homeostasis model assessment of IR; IGF-1 SDS, IGF-1 standard deviation scores; IGF-1, insulin-like growth factor 1; LDL-C, low-density lipoprotein cholesterol; SBP, systolic blood pressure; TC, total cholesterol; TG, triglycerides; UA, uric acid.
